# Hierarchical Encoder-Decoder With Soft Label-Decomposition for Mitochondria Segmentation in EM Images

**DOI:** 10.3389/fnins.2021.687832

**Published:** 2021-06-24

**Authors:** Zhengrong Luo, Ye Wang, Shikun Liu, Jialin Peng

**Affiliations:** ^1^College of Computer Science and Technology, Huaqiao University, Xiamen, China; ^2^School of Statistics, Huaqiao University, Xiamen, China

**Keywords:** image segmentation, convolutional neural networks, electron microscopy image, hierarchical encoder-decoder, mitochondria segmentation

## Abstract

Semantic segmentation of mitochondria from electron microscopy (EM) images is an essential step to obtain reliable morphological statistics about mitochondria. However, automatically delineating plenty of mitochondria of varied shapes from complex backgrounds with sufficient accuracy is challenging. To address these challenges, we develop a hierarchical encoder-decoder network (HED-Net), which has a three-level nested U-shape architecture to capture rich contextual information. Given the irregular shape of mitochondria, we introduce a novel soft label-decomposition strategy to exploit shape knowledge in manual labels. Rather than simply using the ground truth label maps as the unique supervision in the model training, we introduce additional subcategory-aware supervision by softly decomposing each manual label map into two complementary label maps according to mitochondria's ovality. The three label maps are integrated with our HED-Net to supervise the model training. While the original label map guides the network to segment all the mitochondria of varied shapes, the auxiliary label maps guide the network to segment subcategories of mitochondria of circular shape and elliptic shape, respectively, which are much more manageable tasks. Extensive experiments on two public benchmarks show that our HED-Net performs favorably against state-of-the-art methods.

## 1. Introduction

**Figure 1:**
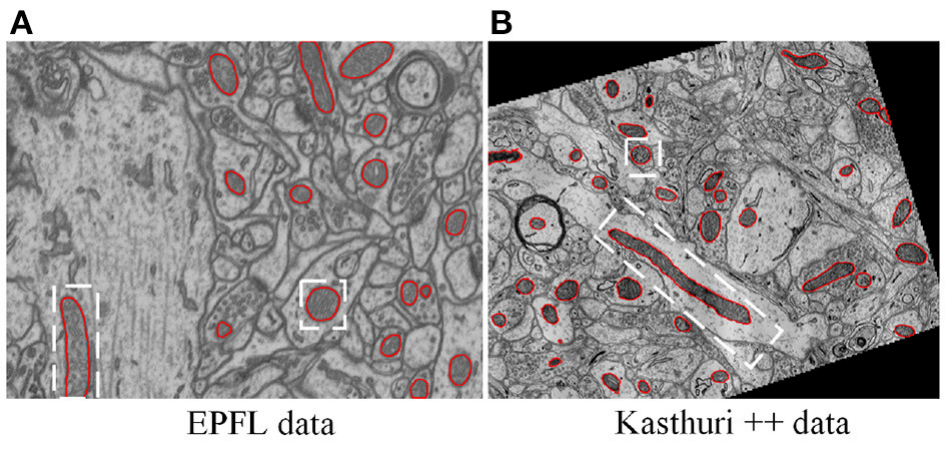
Illustration of typical EM images, in which mitochondria show varied shapes. **(A)** The EPFL data (Lucchi et al., 2013) were taken from CA1 hippocampus region of a mouse brain, **(B)** the Kasthuri++ data (Casser et al., 2020) were taken from mouse cortex. The red contours represent the corresponding ground-truth segmentation.

**Figure 2:**
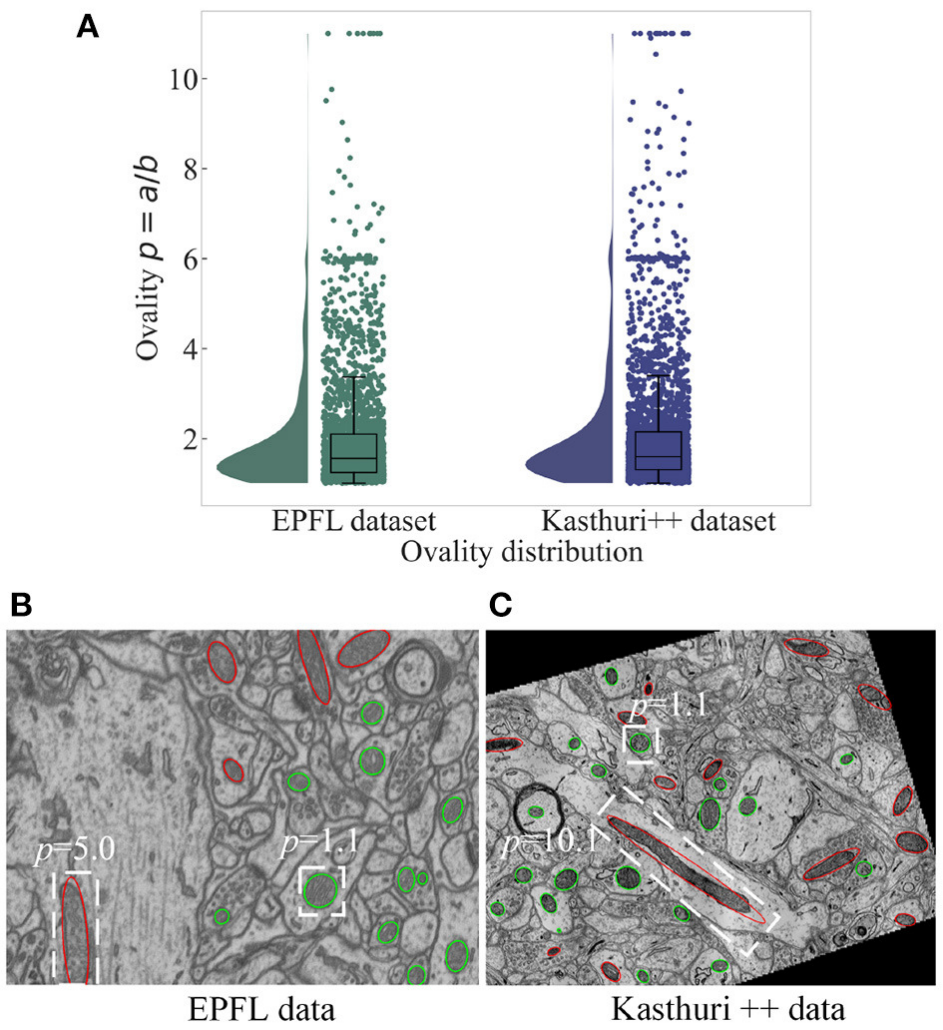
The ovality distribution of mitochondria on images from the EPFL dataset and the Kasthuri++ dataset. The ovality *p* of each mitochondrion is defined as the ratio of the length of its major axis *a* to the length of its minor axis *b*, which can be obtained by performing ellipse fitting on the label maps. The median of ovality *p* distribution **(A)** is 1.56 on the EPFL dataset and 1.60 on the Kasthuri++ dataset. The red contours in **(B,C)** represent mitochondria with *p* over 1.60, and the green contours represent those with *p* lower than 1.60.

Mitochondria are the site of oxidative metabolism in eukaryotes and an essential place to synthesize adenosine triphosphate (ATP) to provide power for cells (Brand et al., 2013). The latest research (Seo et al., 2019) has found that mitochondria are closely related to the occurrence of genetic diseases and the survival of cancer cells. The changes in mitochondrial morphology have a direct impact on the normal realization of their functions. Mitochondria delineation in Electron Microscopy (EM) images plays a vital role in assisting neuroscientists to analyze mitochondrial morphology and distribution of mitochondria. However, manual delineation of mitochondria in many high-resolution EM images requires a vast amount of time and effort by annotation experts. Therefore, automated mitochondria segmentation algorithms with sufficient accuracy are highly desirable to help neurologists analyze EM images. However, mitochondria have varied shapes, ranging from punctuating structures to tubular networks (Wei et al., 2020). Therefore, accurately segmenting mitochondria from complex backgrounds is challenging. Example slices of EM images from two datasets are shown in Figure 1. where mitochondria show irregular shapes and other subcellular structures in the background show similar appearance and shape with mitochondria. Significantly, mitochondria show large variance in roundness. Figure 2A illustrates the ovality distribution of mitochondria on images from the EPFL dataset (Lucchi et al., 2013) and the Kasthuri++ dataset (Casser et al., 2020). The ovality *p* of each mitochondrion is defined as the length *a* of its major axis over the length *b* of its minor axis. The median of ovality *p* distribution is 1.56 on the EPFL dataset and 1.60 on the Kasthuri++ dataset. Further illustrations are shown in Figures 2B,C. in which we conducted ellipse fitting for each mitochondrion instance. It can be seen that some mitochondria have *p* over 10, while some others have *p* lower than 1.5. It is challenging for a model to delineate mitochondria of different shapes simultaneously, which motivates us to exploit subcategory information in our learning based segmentation model.

Recently, various methods (Lucchi et al., 2013; Cheng and Varshney, 2017; Cetina et al., 2018; Xiao et al., 2018; Casser et al., 2020; Peng and Yuan, 2020; Yuan et al., 2021) have been introduced to address mitochondria segmentation. According to the features they used, mitochondria segmentation can be categorized into two classes: traditional methods with hand-crafted features (Lucchi et al., 2011, 2013; Cetina et al., 2018; Peng and Yuan, 2020) and deep learning methods with automatically learned features (Cheng and Varshney, 2017; Xiao et al., 2018; Casser et al., 2020; Yuan et al., 2020, 2021). Generally speaking, deep-learning-based methods, especially methods based on fully convolutional neural networks (Ronneberger et al., 2015; Litjens et al., 2017; Shelhamer et al., 2017), show better performance than traditional machine learning and computer vision methods (Lucchi et al., 2013; Cetina et al., 2018; Peng and Yuan, 2020). Since EM images are volumetric data, both 3D models and 2D models have been adopted in each class. Typically, 3D models (Çiçek et al., 2016; Xiao et al., 2018; Yuan et al., 2021) show better performance by taking advantage of full spatial contexts but at the expense of high computational cost; in contrast, 2D models (Ronneberger et al., 2015; Casser et al., 2020) are more computationally efficient but may neglect inter-slice consistency and show inferior performance. However, 2D methods are flexible to process EM images with large slice thickness. In this study, we follow the slice-by-slice segmentation strategy and aim to devise a powerful 2D model in the deep learning framework.

Among the deep-learning-based methods, the 2D U-Net (Ronneberger et al., 2015) and 3D U-Net (Çiçek et al., 2016), typical encoder-decoder networks with skip connections, are strong baseline models for 2D segmentation and 3D segmentation, respectively. Casser et al. (2020) used a modified 2D U-Net with an on-the-fly data augmentation and Z-filtering postprocessing, and their model showed obviously improved performance over 2D U-Net. Cheng and Varshney (2017) improved the 2D/3D U-Net with factorized convolutions and online feature-level augmentations and showed improved results over the 2D/3D U-Net. Xiao et al. (2018) proposed an effective approach using a modified 3D U-Net, a 3D residual convolutional network with deep supervision. Yuan et al. (2021) introduced a lightweight HIVE-Net with state-of-the-art performance. Their method conducted 3D segmentation but essentially with only more computationally-efficient 2D convolutions. An auxiliary centreline detection task is augmented to capture intrinsic shape prior. Given the high computational complexity of 3D networks, we follow the slice-by-slice segmentation strategy with 2D networks. However, due to the ambiguity of mitochondria segmentation, it is challenging to accurately delineate plenty of mitochondria of varied shapes with information from a single image slice.

To address these challenges, we propose a novel hierarchical encoder-decoder network, named HED-Net, with three-level nested encoder-decoder architecture to capture multi-scale contextual features, which are crucial to discriminate objects from complex backgrounds. Inspired by Qin et al. (2020), we used micro U-Nets to substitute standard convolutions, which constitutes the deeper level of encoder-decoder of our HED-Net. To improve the identification of mitochondria of varied shapes, we propose to exploit shape knowledge from manual labels. Note that manual labels are typically just used as pixel-wise supervision on the final output layer during model training. However, the manual label map for each training image contains more global and semantic information that can be explored to boost the segmentation. Although it is impractical to build a statistical shape model as the prior for multi-object segmentation, it is relatively easy to identify the roundness of each mitochondrion. Based on this observation, we take an easy-to-hard strategy for this challenging binary segmentation problem and introduce subcategory information according to the roundness of the mitochondria, i.e., mitochondria of elliptic shape and mitochondria of circular shape. To guide the model training, we construct two auxiliary label maps with a soft label-decomposition strategy, which decomposes the ground truth label map into two complementary label maps. One label map takes higher values on mitochondria of elliptic shape and lower values on mitochondria of elliptic shape; the other label map takes lower values on mitochondria of elliptic shape and higher values on mitochondria of circular shape. All of the three label-maps jointly supervise the proposed HED-Net. To this end, the outer-level of our HED-Net consists of a soft label-decomposition subnet and a label-fusion subnet, both of which are encoder-decoders. A closely-related method is the decompose-and-integrate strategy in Zhang et al. (2019) for multi-class segmentation, where they split multi-class label map into several binary ones. Our method's significant difference is that we address the binary segmentation problem with a novel soft-label decomposition strategy. The underlying observation is that it is impractical to classify mitochondria into elliptic shapes and circular shapes using a hard threshold based on the ovality of mitochondria.

The main contributions of this study can be summarized as follows,

- We propose a soft label-decomposition strategy to exploit side shape information in manual labels.
- A three-level nested encoder-decoder network is introduced to capture rich contextual information and facilitate the facilitate the integration of subcategory-aware supervision.
- Validations on two challenging benchmarks show that the proposed 2D method can achieve competitive performance in terms of class-level and instance-level measures.

The remainder of this paper is arranged as follows. We elaborate on the proposed methods in section 2. We present experiments and results in section 3. Section 4 concludes this study.

## 2. Method

In this section, we describe the proposed HED-Net in details. To segment mitochondria from volumetric EM images, we follow the slice-by-slice segmentation strategy. To capture inter-slice continuity, the proposed model takes 5-adjacent slices as the input but only outputs the prediction for the centering slice of the multichannel input.

### 2.1. Overview of the Proposed Model

**Figure 3:**
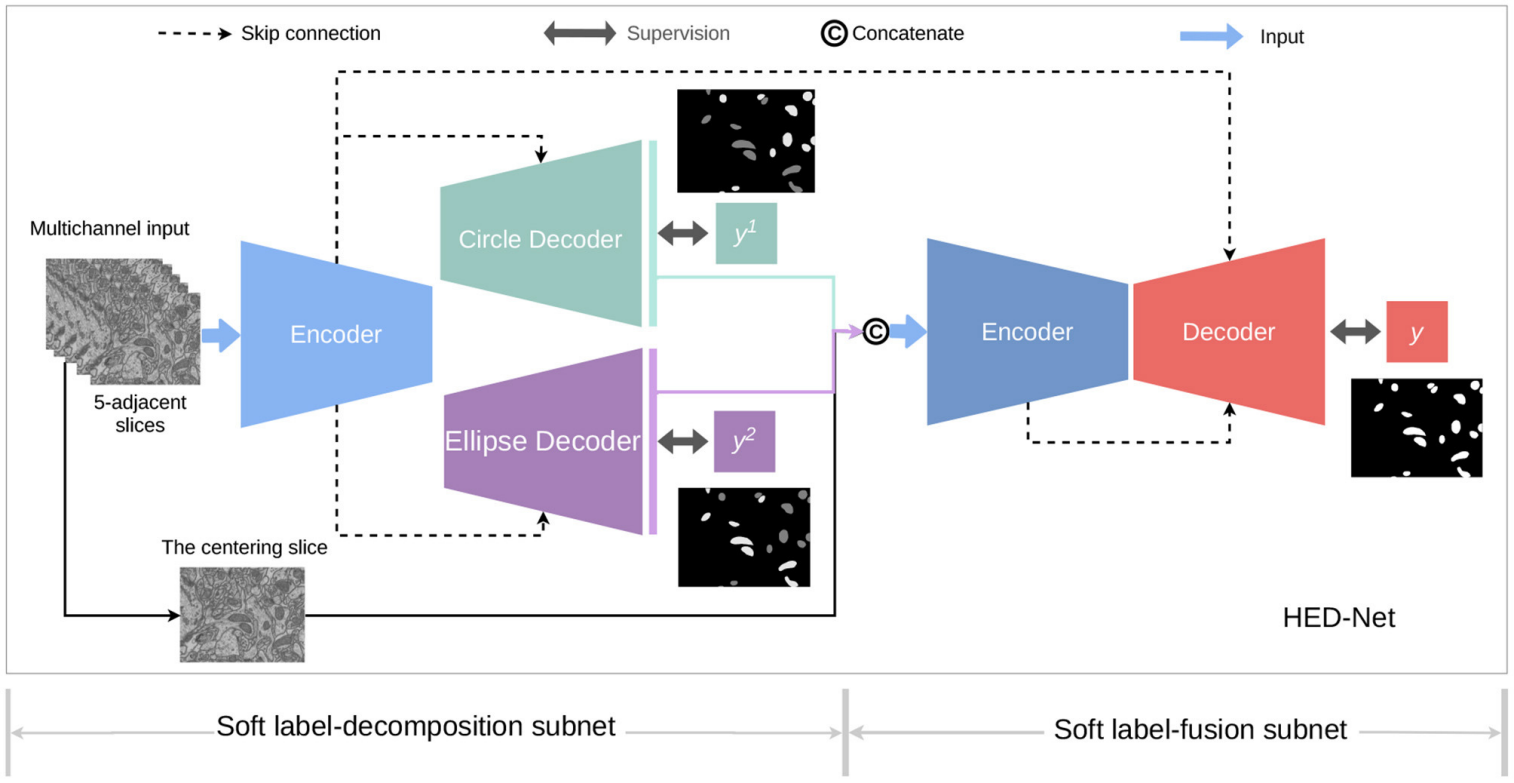
An overview of the proposed HED-Net, which is a three-level nested encoder-decoder with micro U-Nets as the basic building blocks. The HED-Net consists of a soft label-decomposition stage and a soft label-fusion stage. The model is trained under the supervision of ground truth label maps and subcategory-aware label maps.

Figure 3 provides an overview of the proposed HED-Net, which is composed by two stages of encoder-decoder with cross-stage skip-connections and supervised by multiple supervising labels, which will be discussed later in details. The first stage of the HED-Net is a two-head encoder-decoder, named *Soft Label-Decomposition Sub-Net*, which is supervised by auxiliary soft labels for subcategories of mitochondria. The second stage of the HED-Net is an encoder-decoder, named *Soft Label-Fusion Sub-Net*, which fuses the information from the predictions in the first stage and the original images and supervised by original ground truth label map.

In both stages of the HED-Net, we use a slightly modified U-Net with residual connections as the encoder-decoder. The standard U-Net architecture has a contracting path as the encoder to extract semantic features and a symmetric expanding path as the decoder for precise boundary delineation. The standard U-Net used skip-connections between the corresponding encoding and decoding layers. In our model, each encoder has four down-sampling layers including one strided 3 × 3 convolution layer (the first layer) and three 2 × 2 max-pooling layers, and each decoder has four bilinear up-samplings. In terms of architecture, the main differences of the used U-Net in this paper and the standard U-Net (Ronneberger et al., 2015) are the using of strided 3 × 3 convolution for downsampling and bilinear interpolation for unsampling. Moreover, we use residual connections to achieve residual learning (He et al., 2016).

To capture rich contextual features, we replace standard 3 × 3 convolutional layers with micro U-Nets in all the encoder-decoders in the two stages. Each micro U-Net has three max-pooling layers for down-sampling in the encoder and three bilinear up-sampling layers in the decoder. Note that the idea of using small U-Net as the building blocks in U-Net was firstly introduced in Qin et al. (2020) for salient object detection.

### 2.2. Soft Label-Decomposition Subnet

The significant challenges for mitochondria segmentation are the varied shapes and complicated background in EM images, which typically result in missed detection, false detection, and inaccurate boundary delineation, especially for mitochondria of irregular shape. Typically, the manual labels are just used as pixel-wise supervision on the final output layer during model training. However, the manual label map for each training image contains more global and semantic information that can be explored to boost the segmentation of mitochondria. Therefore, we explore general shape knowledge extracted from label maps as side information to improve the segmentation. Although it is impractical to build a statistical shape prior for simultaneously segmenting plenty of mitochondria with varied shape from each EM image, it is relatively easy to identify the roundness of each mitochondrion. Based on this observation, we take an easy-to-hard strategy for this challenging binary segmentation problem and introduce subcategory information according to the roundness of the mitochondria, i.e., mitochondria of elliptic shape and mitochondria of circular shape. The two decoders in the Soft Label-Decomposition Sub-Net focus on the segmentation of mitochondria belongs to the two subcategories, respectively.

#### 2.2.1. Soft Label-Decomposition

**Figure 4:**
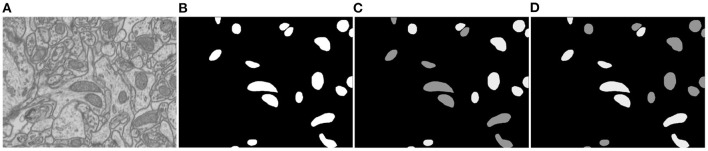
Illustration of the proposed soft label-decomposition. A ground truth label map *Y* in the training data is decomposed into two complementary label maps *Y*^1^ and *Y*^2^ according to roundness of each mitochondrion instance. All the three label maps are jointly used to supervise the model training. **(A)** EM image, **(B)** full label map *Y*, **(C)** circular label map *Y*^1^, **(D)** oval label map *Y*^2^.

Since there is ambiguity to define mitochondria of elliptic shape and mitochondria of circular shape, we introduce a soft label-decomposition strategy to construct auxiliary label maps for supervising the two decoders in the Soft Label-Decomposition Sub-Net. Specifically, given the label map *Y* of a training image *X*, we construct two auxiliary label maps *Y*^1^ and *Y*^2^ according to the roundness of each mitochondrion in *Y*. Suppose *a* is the length of the major axis of a mitochondrion instance, and *b* is the length of the minor axis. We measure the roundness of each mitochondrion instance in *Y* by *p* = *a*/*b*, where the major axis and minor axis are estimated by ellipse fitting, as shown in Figure 2. The mitochondria in Figure 2 show significantly difference in roundness. When *p* approaching 1, the mitochondrion instance is more like a circle. With a given threshold on *p*, we can categorize the mitochondria into two sub-classes, mitochondria of circular shape and mitochondria of elliptical shape, as shown in Figure 4. The two auxiliary label maps are defined as:

(1) $$\begin{array}{l} Y^{1}\left(x\right)=\{\begin{array}{cc} \alpha , & p\left(x\right)\leq T\\ 1-\alpha , & p\left(x\right)>T \end{array}, \end{array}$$

(2) $$\begin{array}{l} Y^{2}\left(x\right)=\{\begin{array}{cc} 1-\alpha , & p\left(x\right)\leq T\\ \alpha , & p\left(x\right)>T \end{array}, \end{array}$$

(3) $$\begin{array}{l} Y=Y^{1}+Y^{2}, \end{array}$$

where *x* is a pixel in *X*, *p*(*x*) is the roundness of the mitochondrion instance that *x* belongs to, α ∈ [0.5, 1] is a positive constant, and the ovality threshold *T* is a positive value. With α ∈ (0.5, 1], the label maps *Y*^1^ and *Y*^2^ put unequal weights on mitochondria of different shapes. In our experiments, the parameter *T* is set as 1.6 according to the mitochondrion instance's statistical distribution in the training dataset. We set α=0.9 to make the two label maps *Y*^1^ and *Y*^2^ highlight mitochondria of different subcategories. Although we can also achieve a hard label-decomposition by setting set α = 1, it is challenging for a segmentation model to identify mitochondria in each subcategory.

#### 2.2.2. Subcategory-Aware Supervision

The two auxiliary label maps *Y*^1^ and *Y*^2^ are used as deep supervision to guide the model training. The soft label-decomposition subnet consists of two decoders: a circular decoder that is mainly responsible for detecting circular mitochondria, and an ellipse decoder that is mainly responsible for detecting oval-shaped mitochondria. Since there is ambiguity on the class boundary between circular mitochondria and oval-shaped mitochondria, each decoder segments all mitochondria but puts higher weights on its focused subcategory.

### 2.3. Soft Label-Fusion Subnet

To integrate the predictions and features of the soft label-decomposition stage, we introduce a soft label-fusion stage, which is supervised by full ground truth labels and jointly trained with the first stage. The soft label-fusion subset takes the predictions of the first stage and the original image as input. The features in the encoders of the first stage is reused in the decoder of the second stage with skip-connections and concatenation. The soft label-decomposition subnet and the soft label-fusion subnet constitute the proposed HED-Net.

### 2.4. The Total Loss

Let *P*^1^, *P*^2^, and *P* be the predictions of the circle decoder, the ellipse encoder, and the decoder of the second stage, respectively. The total loss of our HED-Net is defined as following,

(4) $$\begin{array}{l} L_{total}=\lambda L_{circle}+\lambda L_{ellipse}+L, \end{array}$$

where λ is a positive trade-off parameter. *L*_*circle*_, *L*_*ellipse*_, and *L* denotes the losses for the circle decoder, the ellipse encoder, and the decoder of the second stage. They are defined based on the Dice loss function,

(5) $$\begin{array}{l} Dice\left(P,Y\right)=1-\frac{2\sum_{x}Y\left(x\right)P\left(x\right)}{\sum_{x}Y\left(x\right)Y\left(x\right)+\sum_{x}P\left(x\right)P\left(x\right)+\epsilon }, \end{array}$$

where *x* is a pixel location.

It is noteworthy that, since the auxiliary label maps *Y*^1^ and *Y*^2^ take different values on different mitochondrion instances, *Dice*(*P*^1^, *Y*^1^) and *Dice*(*P*^2^, *Y*^2^) are essentially *weighted Dice losses*.

## 3. Results and Analysis

In this section, we first evaluate the segmentation and detection performance of our method on two public benckmarks, and then conduct an ablation analysis of our model.

### 3.1. Datasets

We evaluate model performance on two mitochondria datasets, which have different voxel spacings and different volume sizes. The public EPFL dataset^1^ provides two stacks for model training and testing, respectively; each stack has 165 consecutive slices of size 768 × 1,024, which were scanned with focused ion beam scanning EM (FIBSEM) from CA1 hippocampus region of a mouse brain. Kasthuri++ dataset^2^ contains 85 consecutive image slices of size 1,643 × 1,613 for model training and 75 slices of size 1,334 × 1,553 for model testing. The images in Kasthuri++ dataset were taken from 3-cylinder mouse cortex with serial section EM (ssEM). The Kasthuri++ dataset was relabeled by Casser et al. (2020). The two datasets have significantly different voxel spacing. While the voxel spacing of EPFL dataset is 5 *nm*^3^ per voxel and the voxel spacing of Kasthuri++ dataset is 3 × 3 × 30 *nm* per voxel.

### 3.2. Evaluation Criteria

#### 3.2.1. Criteria for Evaluating Binary Segmentation

Dice similarity coefficient (DSC) and Jaccard-index coefficient (JAC) are used to measure the agreement between the binary ground truth *Y* and predicted segmentation *P*.

(6) $$\begin{array}{l} \text{DSC}=\frac{2|P\cap Y|}{\left|P|+|Y\right|},\text{JAC}=\frac{\left|P\cap Y\right|}{\left|P\cup Y\right|}. \end{array}$$

#### 3.2.2. Criteria for Evaluating Instance Segmentation

We use the aggregated Jaccard-index (AJI) (Kumar et al., 2017) and Panoptic Quality (PQ) (Graham et al., 2019; Kirillov et al., 2019) to evaluate the instance segmentation performance.

(7) $$\begin{array}{l} \text{AJI}=\frac{\sum_{j=1}^{N}|Y^{j}\cap P^{j^{*}}|}{\sum_{j=1}^{N}|Y^{j}\cup P^{j^{*}}|+\sum_{i\in \text{FP}}|P^{i}|}, \end{array}$$

where *N* is the total number of instance in *Y*, *P*^j^*^^ is the segment (i.e., connected region) in the predicted segmentation that has the largest overlapping (in terms of JAC) with the segment *Y*^*j*^; FP is the set of false positive regions in *P* without the matched mitochondria in *Y*.

(8) $$\begin{array}{l} PQ=\underset{\text{Segmentation Quality}(\text{SQ})}{\underset{︸}{\frac{\sum_{j\in \text{TP}}\text{JAC}(Y^{j},P^{j^{*}})}{\left|\text{TP}\right|}}}\times \underset{\text{Detection Quality}(\text{DQ})}{\underset{︸}{\frac{\left|\text{TP}\right|}{\left|\text{TP}|+\frac{1}{2}|\text{FP}|+\frac{1}{2}|\text{FN}\right|}}}, \end{array}$$

where true positives (TP), false positives (FP), and false negatives (FN) representing the matched pairs of segments with at least 50% overlapping in JAC, unmatched predicted segments, and unmatched ground truth segments, respectively.

#### 3.2.3. Criteria for Evaluating Detection

By default, we use F1-75, which requires at least 75% overlap in JAC. Moreover, given the TP and FN, we also report the sensitivity (SEN) and specificity (SPE).

(9) $$\begin{array}{l} \text{SEN}=\frac{\left|\text{TP}\right|}{\left|\text{TP}|+|\text{FN}\right|},\text{ SPE}=\frac{\left|\text{TN}\right|}{\left|\text{TN}|+|\text{FP}\right|}. \end{array}$$

The detection performance in F1 under different overlapping requirements (50–85%) are also used. Especially, F1-80 and F1-85 are very strict measures.

### 3.3. Implementation Details

We use Pytorch (Paszke et al., 2019) on a workstation with 64 GB RAM and one GTX 2080Ti GPU to implement our experiments. The trade-off parameter λ is fixed and set as 0.5 to make the training losses of the first stage and the second stage have the similar magnitudes. Thus, the two stages have the same importance. The model is optimized by Adam (Kingma and Ba, 2014), and the weight decay is set to 10^−5^. The initial learning rate is set as 5 × 10^−4^ and a step-wise learning rate decay scheme is employed. For the EPFL dataset, the step and decay rate is set to 30 and 0.9, respectively; For the Kasthuri++ dataset, the step and decay rate is set as 60 and 0.9, respectively. Our network is trained using randomly cropped images of size 512 × 512 and batch size 3 for all the two datasets. Synthesized images and the corresponding label maps through flipping, gaussian blur, median blur, and random rotations of ±90° are used as data augmentation to the training data. At the inference time, we apply the test-time argumentation, including flipping and rotation of ±90° to improve the performance further.

### 3.4. Segmentation Performance

We compare our method with both 2D methods and 3D methods, including both traditional methods based on hand-crafted features (Lucchi et al., 2013; Cetina et al., 2018; Peng and Yuan, 2020) and deep learning methods (Ronneberger et al., 2015; Çiçek et al., 2016; Cheng and Varshney, 2017; Xiao et al., 2018; Casser et al., 2020), on the EPFL dataset and Kasthuri++ dataset. Since our HED-Net takes 5-slice input, which is usually called 2.5D method, we also compare our method with 2D U-Net (Ronneberger et al., 2015) that takes five slices as input.

#### 3.4.1. Visual Comparison

**Figure 5:**
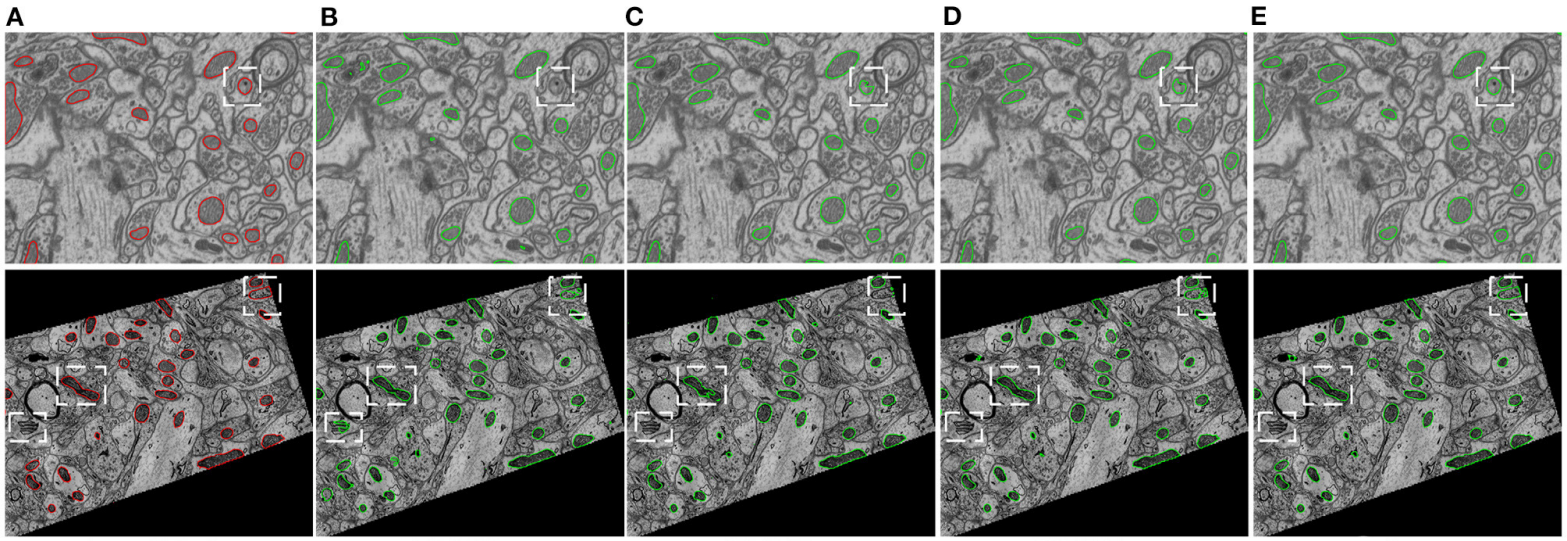
Visual comparison of the proposed method with two strong baselines, i.e., 2D U-Net (Ronneberger et al., 2015) and 3D U-Net (Çiçek et al., 2016) and a state-of-the-art 3D model, i.e., HIVE-Net (Yuan et al., 2021), on examples from the EPFL dataset and the Kasthuri++ dataset. **(A)** Ground truth, **(B)** 2D U-Net, **(C)** 3D U-Net, **(D)** HIVE-Net, **(D)** our.

Figure 5 provides visual comparisons of the proposed method with two strong baselines, i.e., 2D U-Net (Ronneberger et al., 2015) and 3D U-Net (Çiçek et al., 2016) and a state-of-the-art 3D model, i.e., HIVE-Net (Yuan et al., 2021), on examples in EPFL dataset and Kasthuri++ dataset. In comparison of the results in Figures 5B,C,E, we can see that the proposed method obviously shows fewer false detections and fewer missed detections than 2D U-Net and 3D U-Net. As shown in Figures 5D,E, the proposed 2D model shows comparable visual performance with the 3D model HIVE-Net but with slightly better shape integrity.

#### 3.4.2. Segmentation Performance on EPFL Dataset

Table 1 demonstrates the quantitative comparison of our method with both 3D methods, 2.5D methods, and 2D methods for mitochondria segmentation on the EPFL dataset. While the methods in Lucchi et al. (2013), Cetina et al. (2018), and Peng and Yuan (2020) are traditional methods with handcrafted features, other methods are deep learning based methods, which show better results than traditional methods. Compared to 2D methods that take single slice as input, 2.5D methods takes multiple slices as input. With more slices as input, the U-Net (5-slice input) outperforms the U-Net (1-slice input) by 1.4% in DSC. The 3D U-Net and methods in Cheng and Varshney (2017), Xiao et al. (2018), and Yuan et al. (2021) directly segment 3D volumes and generally show better results than 2D methods and 2.5D methods. The HIVE-Net shows the best results among the 3D models. However, 3D models usually suffer from high computational complexity.

**Table 1:** Comparison of the proposed method with other 2D/2.5D/3D top-performing methods for mitochondria segmentation on EPFL dataset.

**Type**	**Method**	**Binary seg**.		**Instance seg**.	
		**DSC**	**JAC**	**AJI**	**PQ**
3D	Lucchi et al. (2013)	86.0	75.5	74.0	63.5
	Cetina et al. (2018)	86.4	76.0	–	–
	Peng and Yuan (2020)	90.9	83.3	75.4	67.7
	3D U-Net (Çiçek et al., 2016)	93.5	87.8	86.9	80.6
	Cheng and Varshney (2017) (3D)	94.1	88.9	–	–
	Xiao et al. (2018)	94.7	90.0	88.6	83.1
	HIVE-Net (Yuan et al., 2021)	**94.8**	**90.1**	89.0	83.9
2D	U-Net (1-slice input)	91.5	84.4	83.0	75.5
	Cheng and Varshney (2017) (2D)	92.8	86.5	–	–
	Casser et al. (2020) (w/o Z-Filtering)	93.8	88.4	88.0	81.5
	Casser et al. (2020) (w Z-Filtering)	94.2	89.0	88.5	83.0
	HED-Net (1-slice input)	94.2	89.1	89.1	84.0
2.5D	U-Net (5-slice input)	92.9	86.8	86.6	78.7
	HED-Net	94.7	89.9	**89.7**	**85.0**

*The evaluation results under measures (%) for binary segmentation and instance segmentation*.

*Best results are highlighted in bold*.

From the Table 1, we can see that the proposed HED-Net not only shows the best segmentation performance among all the 2D models and 2.5D models, but also shows competitive performance in comparison with 3D models. Especially, for instance segmentation, our model outperforms the HIVE-Net by 0.7 and 1.1% in terms of AJI and PQ, respectively. For binary segmentation, the performance of our method is only slightly lower (≤0.2%) than the HIVE-Net. When taking single slice as the input, our HED-Net (1-slice) outperforms most of the compared methods except for Xiao et al. (2018) and HIVE-Net (Yuan et al., 2021), and show similar performance as Casser et al. (2020), who used a median filter along the z-dimension (Z-Filtering) as post-processing to capture 3D information.

#### 3.4.3. Segmentation Performance on Kasthuri++ Dataset

Table 2 demonstrates the quantitative comparison of the performances of different methods for mitochondria segmentation on the Kasthuri++ dataset. The proposed method shows significant improvements over the strong baseline models, i.e., 2D U-Net (5-slice) and 3D U-Net and obtains 96.1% in DSC for binary segmentation, 91.6% in AJI, and 86.6 in PQ for instance segmentation, outperforming the 3D U-Net by 1.8% in DSC, 3.4% in JAC, 3.7% in AJI, and 5.1% in PQ. The proposed model outperforms the method in Xiao et al. (2018) by 0.2% in DSC, 0.4% in JAC, 0.6% in AJI, and 1.5% in PQ. The proposed model shows competitive performance in comparison with the HIVE-Net but is flexible to process both 2D and 3D data.

**Table 2:** Comparison of the proposed method with other 2D/3D top-performing methods for mitochondria segmentation on Kasthuri++ dataset.

**Type**	**Method**	**Binary seg**.		**Instance seg**.	
		**DSC**	**JAC**	**AJI**	**PQ**
3D	Lucchi et al. (2013)	86.2	75.8	73.5	57.6
	Peng and Yuan (2020)	89.3	80.6	85.8	72.9
	3D U-Net (Çiçek et al., 2016)	94.3	89.2	87.9	81.5
	Xiao et al. (2018)	95.9	92.2	91.0	85.1
	HIVE-Net (Yuan et al., 2021)	**96.2**	**92.8**	91.5	**86.6**
2D	U-Net (1-slice input)	94.0	88.6	87.5	80.2
	Casser et al. (2020) (w/o Z-Filtering)	91.5	84.4	83.5	77.8
	Casser et al. (2020) (w Z-Filtering)	89.4	81.0	78.3	71.6
	HED-Net (1-slice input)	95.9	92.2	91.3	85.1
2.5D	U-Net (5-slice input)	94.4	89.3	88.1	81.6
	HED-Net	96.1	92.6	**91.6**	**86.6**

*The evaluation results under measures (%) for binary segmentation and instance segmentation*.

*Best results are highlighted in bold*.

#### 3.4.4. Detection Performance

The detection performance is also crucial for evaluating the proposed method. To this end, we compare our method with other methods in terms of F1-75, SPE, and SEN. Table 3 summarizes the quantitative comparison results on both the EPFL dataset and the Kasthuri++ dataset. Overall, our method shows the best performance on both of the two datasets and outperforms the 2D U-Net by 11.0 and 6.5% in F1-75 on the EPFL dataset and the Kasthuri++ dataset, respectively. Moreover, the proposed 2D model outperforms the state-of-the-art HIVE-Net by 1.9 and 1.2% in F1-75 on the EPFL dataset and the Kasthuri++ dataset, respectively. Significantly, our method shows higher specificity, which indicates that our model has strong ability to control false detection. These results demonstrate the effectiveness of our method.

**Table 3:** Detection performance on EPFL and Kashuri++.

**Type**	**Method**	**EPFL**			**Kashuri++**		
		**F1-75**	**SEN**	**SPE**	**F1-75**	**SEN**	**SPE**
3D	Lucchi et al. (2013)	42.0	45.3	39.5	57.0	64.0	52.0
	Peng and Yuan (2020)	75.7	79.8	72.3	70.3	68.8	72.2
	3D U-Net (Çiçek et al., 2016)	87.7	89.8	86.0	84.9	85.5	84.4
	Xiao et al. (2018)	87.8	89.0	87.0	87.2	87.4	87.2
	HIVE-Net (Yuan et al., 2021)	90.1	91.2	89.3	89.1	**89.6**	88.8
2D	U-Net (1-slice input)	81.0	85.3	77.6	83.8	82.9	85.1
	Casser et al. (2020) (w Z-Filtering)	89.8	90.5	89.6	73.9	72.4	75.8
2.5D	U-Net (5-slice input)	84.4	88.3	81.7	85.6	85.2	86.4
	HED-Net	**92.0**	**92.1**	**92.2**	**90.3**	87.1	**93.9**

*Evaluation results under F1-75, SEN, and SPE are reported which are based on measuring the segment overlapping of matched instances*.

*Best results are highlighted in bold*.

**Figure 6:**
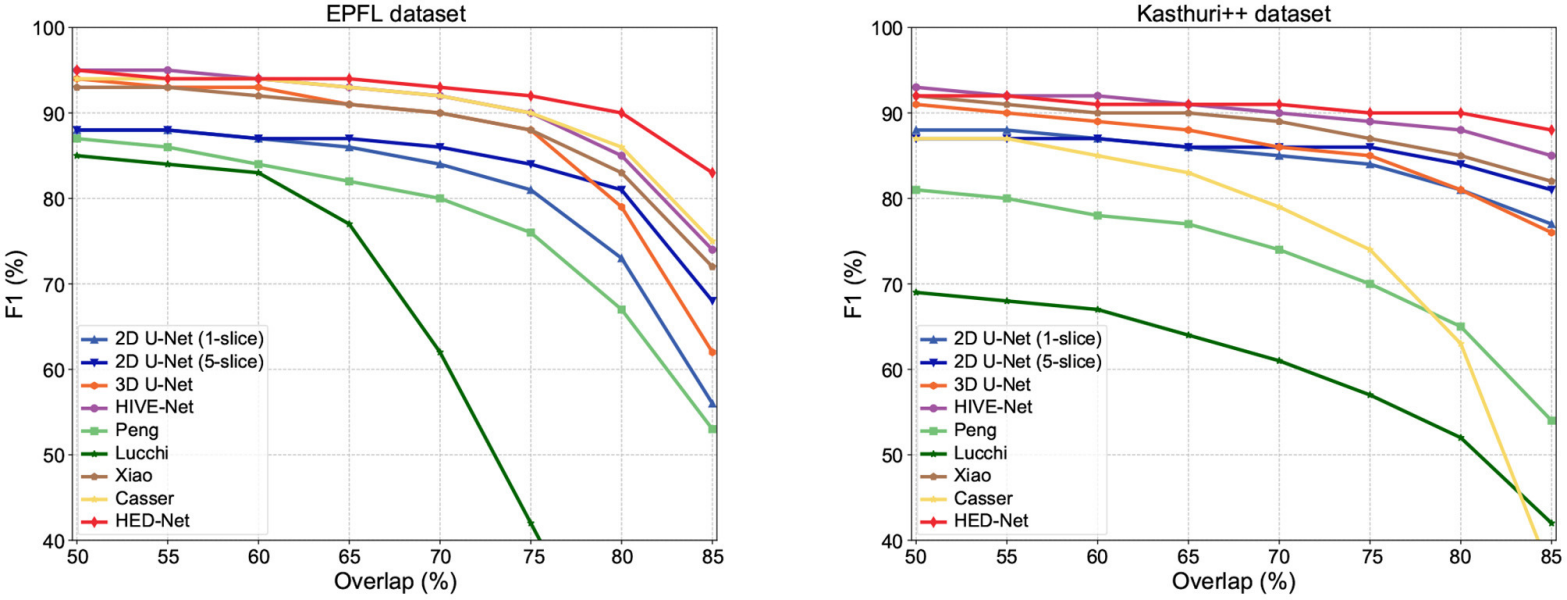
Detection performance in F1 with different overlapping thresholds for matched instances. As the overlapping thresholds increase, the F1 of all methods has decreased, but our method maintains the highest stability.

In addition to F1-75, we compare our method with other methods in terms of F1 values that use other overlapping requirements. The comparison results are illustrated in Figure 6. Our model shows superior performance over other methods, especially in terms of F1-80 and F1-85, which are very strict detection measures. Therefore, these results also indicate that the segmentation by our proposed model can match the ground truth segmentation better.

### 3.5. Impact of the Number of Input Slices

We first study the impact of using varying input slices on segmentation performance, which is demonstrated in Table 4. By comparing the results in four measures, we have three conclusions. First, making use of multi-slice input can improve the performance of the U-Net and our HED-Net. However, the performance gain can be marginal when increasing the number of input slices. Second, compared to the U-Net, the proposed HED-Net obtains a smaller performance gain when using multi-slice input. Third, 5-slice input is the best choice for our model. Therefore, by default, we use five neighboring slices as input for our model in the following experiments.

**Table 4:** The impact of using varying number of input slices on the segmentation performance.

**Input slices**	**U-Net**				**HED-Net**			
	**DSC**	**JAC**	**AJI**	**PQ**	**DSC**	**JAC**	**AJI**	**PQ**
1	91.5	84.4	83.0	75.5	94.2	89.1	89.1	84.0
3	92.5	86.2	85.9	77.2	94.5	89.6	89.5	84.9
5	92.9	86.8	**86.6**	78.7	**94.7**	**89.9**	**89.7**	**85.0**
7	**93.0**	**86.9**	**86.6**	**79.0**	94.5	89.7	89.6	84.9

*By default, we use 5-neighboring slices as input. The compared methods are validated on the EPFL dataset*.

*Best results are highlighted in bold*.

### 3.6. Impact of the Tradeoff Parameter λ

We further investigate the impact of the hyper-parameter λ on the segmentation performance of the proposed HED-Net. The hyper-parameter λ trades off the importance of the two stages of the HED-Net. Since the first stage of the HED-Net contains two decoders and the second stage contains only one decoder, we set λ as 0.5 by default to have equal importance on the two stages. In this section, we further test the segmentation performance with other choices of λ, i.e., 0.1, 0.25, and 1.0. The segmentation results are summarized in Table 5. We can see that balanced importance of the two stages with λ=0.5 results in a better performance.

**Table 5:** The impact of the tradeoff parameter λ.

**λ**	**HED-Net**			
	**DSC**	**JAC**	**AJI**	**PQ**
0.10	94.4	89.4	89.3	84.0
0.25	94.5	89.6	89.7	85.3
0.50	**94.7**	**89.9**	**89.7**	**85.0**
1.00	94.3	89.3	89.1	84.1

*The results are the performance on the EPFL dataset*.

*Best results are highlighted in bold*.

### 3.7. Ablation Study

**Figure 7:**
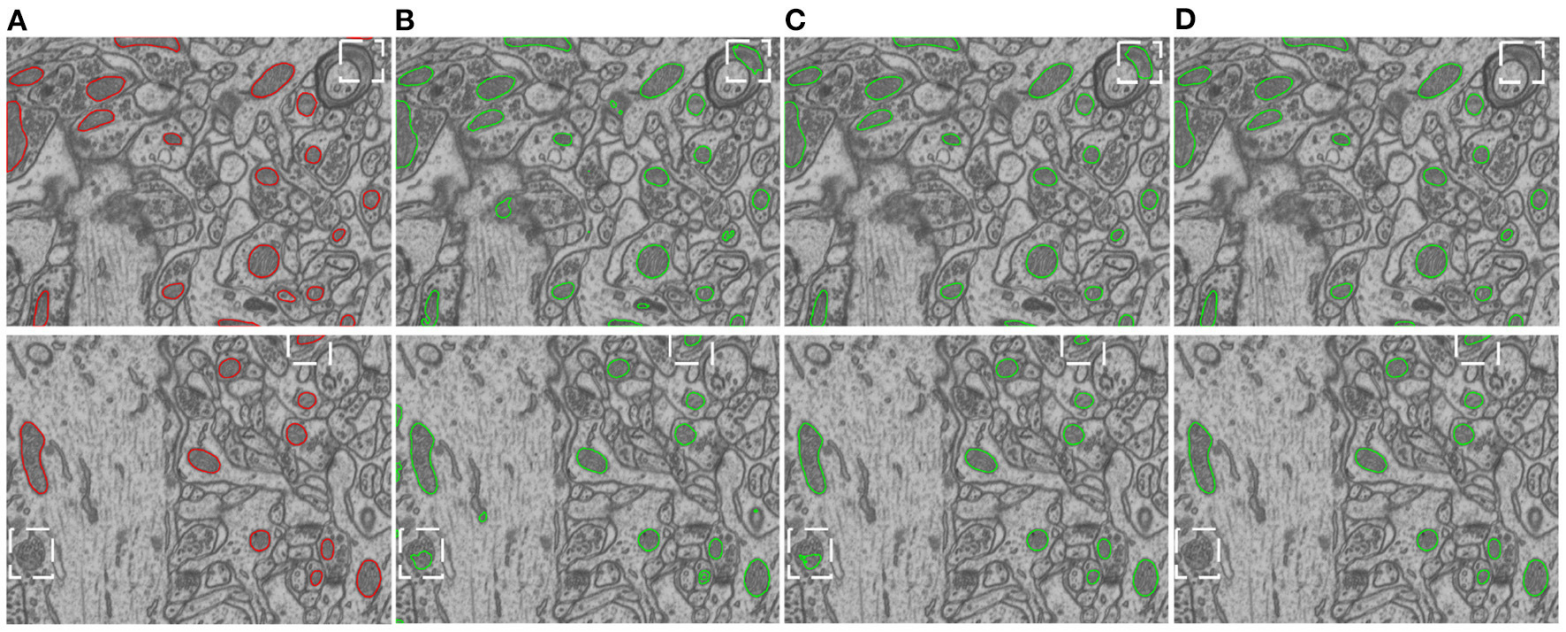
Visual comparison of our model with its ablated versions on the EPFL dataset. **(A)** Ground truth, **(B)** 2D U-Net, **(C)** HED-Net w/o label-decompn, **(D)** HED-Net.

**Figure 8:**
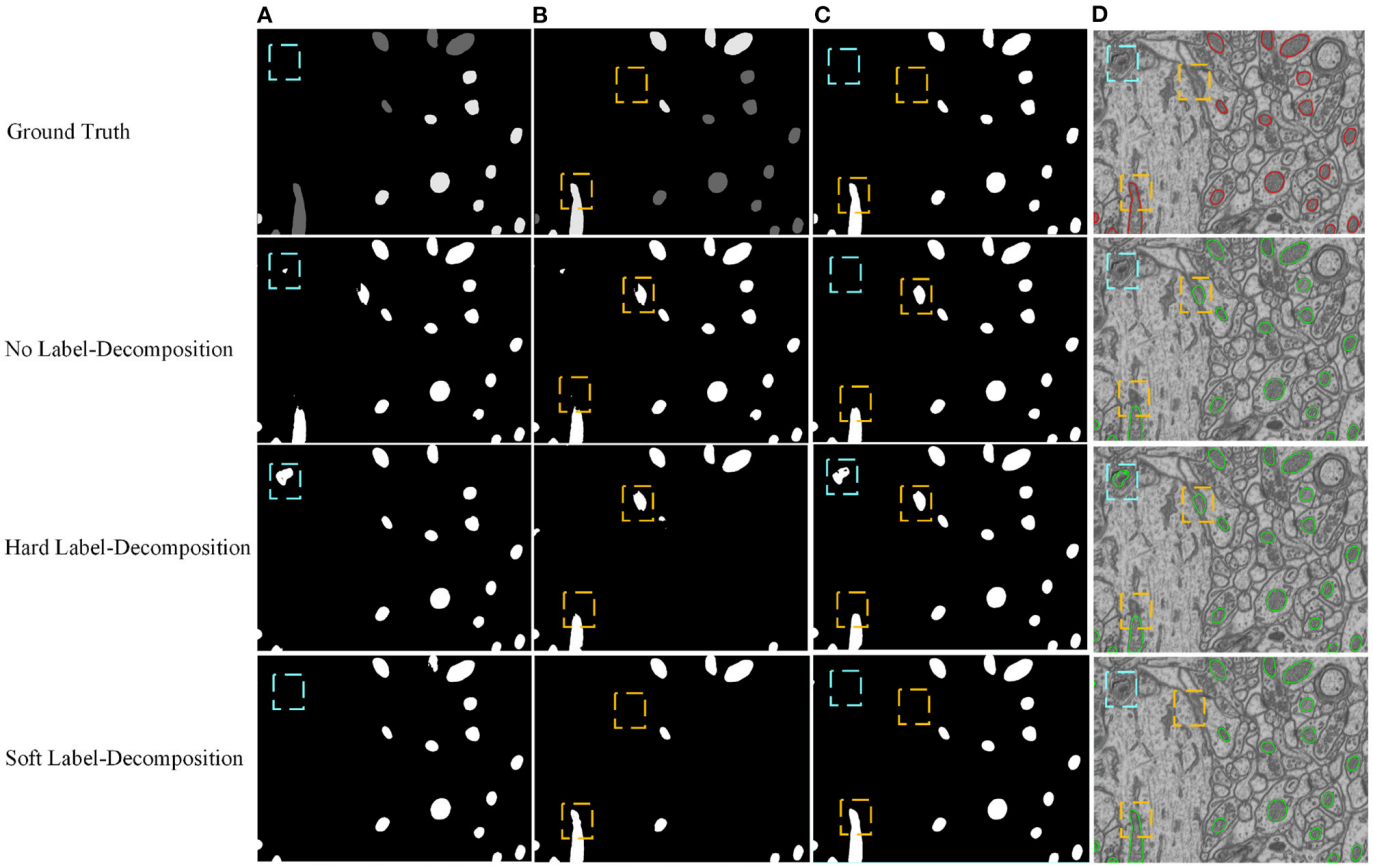
Visual comparison of predictions of the circle decoder and ellipse decoder in the first stage and the fusion decoder in the second stage. The results of the HED-Net with no label-decomposition, hard label-decomposition, and soft label-decomposition are reported. **(A)** Circle decoder pred., **(B)** ellipse decoder pred., **(C)** fusion decoder pred., **(D)** overlaid segmentation.

We conduct ablation studies to identify the effectiveness of the introduced two-stage network architecture, the impact of using micro U-Net as building blocks, and the superiority of the proposed soft label-decomposition strategy. Table 6 illustrates the performance of the proposed HED-Net under different network settings. The last row is our complete model, which uses two-stage network architecture with micro U-Net as the basic building block and utilizes the soft label-decomposition strategy to achieve subcategory-aware learning. As can be seen, the models using one-stage architecture are overall poor than the one using the two-stage architecture. Significantly, when using standard 3 × 3 convolution as the basic building block, the two-stage network outperforms the one-stage network by a large margin for both binary segmentation and instance segmentation. Moreover, the using of micro U-Net in the two-stage HED-Net results in a performance gain of 0.6% in DSC, 1.1% in JAC, 1.1% in AJI, and 4.6% in PQ, which indicate its effectiveness. Furthermore, with the soft label-decomposition, we obtain a performance gain of 0.6% in DSC, 1.0% in JAC, 1.0% in AJI, and 1.3% in PQ. Compared to the HED-Net using hard label-decomposition, the HED-Net using soft label-decomposition shows superior performance. A visual comparison of the complete HED-Net with the HED-Net without using label-decomposition and the U-Net are shown in Figure 7. The results of our HED-Net shows much fewer false detections and more accurate boundary delineations. Figure 8 provides further visual comparison of predictions of the circle decoder and ellipse decoder in the first stage and the fusion decoder in the second stage. The segmentation results by the HED-Net with no label-decomposition, hard label-decomposition, and soft label-decomposition are illustrated. The results of the circle decoder and ellipse decoder of the HED-Net are complementary. Compared to the HED-Net with no label-decomposition and with hard label-decomposition, the HED-Net with soft label-decomposition shows reduced false positives and a stronger ability to capture mitochondria of large ovality.

**Table 6:** Ablation study of the proposed HED-Net on EPFL dataset.

**Methods**	**Architecture**	**Convolutions**	**Label decomposition**	**DSC**	**JAC**	**AJI**	**PQ**
HED-Net	One stage	3 × 3 Conv.	–	92.9	86.8	86.6	78.7
	One stage	Micro U-Net	–	93.9	88.7	88.6	83.1
	Two stages	3 × 3 Conv.	–	93.5	87.8	87.6	79.1
	Two stages	Micro U-Net	–	94.1	88.9	88.7	83.7
	Two stages	Micro U-Net	Hard (α = 1.0)	94.4	89.5	89.5	84.5
	Two stages	Micro U-Net	Soft (α = 0.9)	**94.7**	**89.9**	**89.7**	**85.0**

*The complete HED-Net uses two-stage network architecture with micro U-Net as the basic building block and utilizes the soft label-decomposition strategy to achieve subcategory-aware learning. All the methods in comparison use five slices as input*.

*Best results are highlighted in bold*.

### 3.8. The Impact of the Ovality Threshold *T*

We have also investigated the impact of choosing different ovality thresholds *T* for the HED-Net with hard and soft label-decomposition. Given the ovality *p* distributions in Figure 2, we choose the median of *p*, i.e., *T* = 1.6, as the default setting. In this section, we test other choices of *T*, i.e., first quartile (*T* = 1.3) and third quartile (*T* = 2.1). The results on the EPFL dataset are reported in Table 7. We can see that, the median of the ovality distribution is a better choice as the ovality threshold than the first quartile and third quartile. The HED-Net with the soft label-decomposition consistently outperforms the HED-Net with the hard label-decomposition.

**Table 7:** The impact of choosing different thresholds *T* for the ovality *p*.

**HED-Net**	**T = 1.3**				**T = 1.6**				**T = 2.1**			
	**DSC**	**JAC**	**AJI**	**PQ**	**DSC**	**JAC**	**AJI**	**PQ**	**DSC**	**JAC**	**AJI**	**PQ**
Hard (α = 1.0)	94.2	89.1	89.0	84.2	94.4	89.5	89.5	84.5	94.0	88.8	88.7	83.9
Soft (α = 0.9)	94.5	89.5	89.5	84.6	94.7	89.9	89.7	85.0	94.4	89.4	89.3	84.8

*The first quartile (T = 1.3), median (T = 1.6), third quartile (T = 2.1) of the ovality distribution are tested. The HED-Net with both hard label-decomposition and soft label-decomposition are evaluated on the EPFL dataset*.

### 3.9. The Inference Time

**Figure 9:**
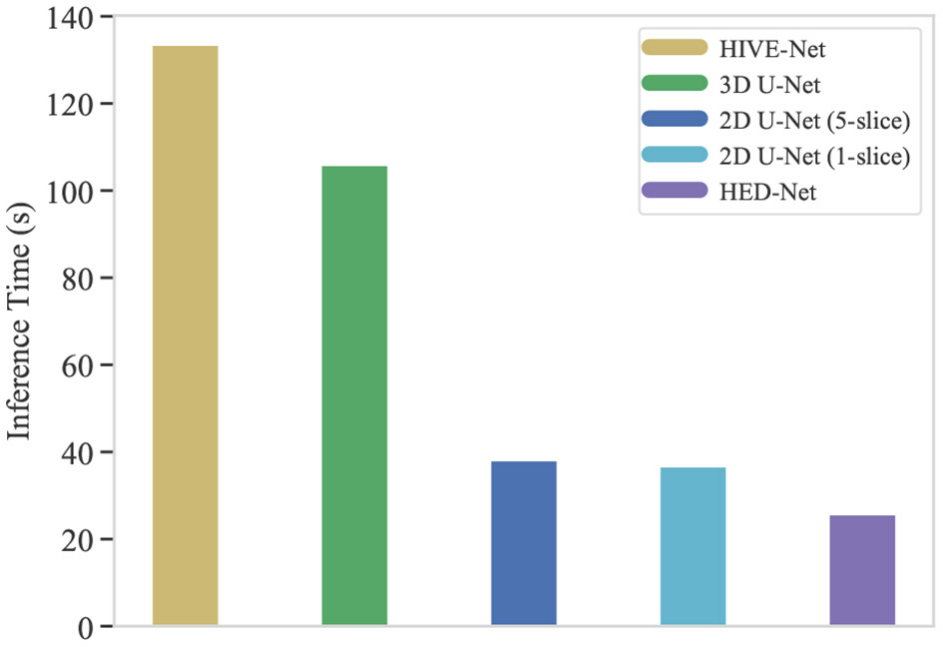
The inference time (in second) of typical segmentation models for 165 images of 768 × 1,024. Our proposed HED-Net takes 25.7 s for inference, while the HIVE-Net takes 133.5 s for inference.

One of the crucial aspects of deploying a segmentation network is the inference time. In this section, we compare the inference time of our HED-Net with other 2D/2.5D/3D methods, i.e., 2D U-Net (1-slice), 2D U-Net (5-slice), 3D U-Net, and HIVE-Net. More specifically, we calculate the total inference time on the testing stack of the EPFL data, which contains 165 consecutive images of size 768 × 1,024. While the 2D and 2.5D methods, i.e., 2D U-Net (1-slice), 2D U-Net (5-slice), and our HED-Net, conduct slice-by-slice segmentation, the 3D U-Net and HIVE-Net segment all the images in one pass. The comparative results are illustrated in Figure 9. It can be seen that our method takes a much shorter inference time than the compared methods. Significantly, our proposed HED-Net takes 25.7 s for the inference of all the testing images, while the top-performing method HIVE-Net takes 133.5 s for inference.

## 4. Conclusions

In this paper, we have proposed a hierarchical encoder-decoder network for mitochondria segmentation from EM images. To address the challenge of the varied shape of mitochondria and complex backgrounds, we followed an easy-to-hard strategy. Specifically, we introduced a novel soft label-decomposition strategy, which resulted in additional subcategory-aware supervision for our model. The proposed network utilized a three-level nested U-shape architecture to capture rich contextual information and employed general shape information in manual labels to reduce missed detection and false detection. The proposed method has been evaluated on two challenging benchmarks. Comparisons with strong baseline models and top-performing 2D/3D methods showed that our method showed state-of-the-art results. Significantly, the proposed model showed superior results for instance segmentation and detection tasks. Ablation studies further demonstrated the effectiveness of the proposed model.

In future work, we will utilize the size attribute and symmetry attribute of the targets as the side information further to explore the global information in the manual label maps. Moreover, we will employ more advanced image synthesis methods (Peng and Wang, 2021) to reduce the amount of labeled data requested for model training.

## Data Availability Statement

The original contributions presented in the study are included in the article/supplementary material, further inquiries can be directed to the corresponding author/s.

## Author Contributions

ZL and YW mainly implemented the method, conducted the experiments, and contributed to the writing of the paper. SL helped perform the analysis with constructive discussions. JP supervised the whole process, including the development of the concept, writing, revision, and other general advice. All authors approved it for publication.

## Conflict of Interest

The authors declare that the research was conducted in the absence of any commercial or financial relationships that could be construed as a potential conflict of interest.

## Footnotes

^1^https://cvlab.epfl.ch/data/em.

^2^https://casser.io/connectomics/.

## References

[B1] Brand M. Orr A. Perevoshchikova I. Quinlan C. (2013). The role of mitochondrial function and cellular bioenergetics in ageing and disease. Br. J. Dermatol. 169, 1–8. 10.1111/bjd.12208 23786614 PMC4321783

[B2] Casser V. Kang K. Pfister H. Haehn D. (2020). Fast mitochondria detection for connectomics, in Medical Imaging With Deep Learning (Montréal, QC).

[B3] Cetina K. Buenaposada J. M. Baumela L. (2018). Multi-class segmentation of neuronal structures in electron microscopy images. BMC Bioinformatics 19:298. 10.1186/s12859-018-2305-0 30092759 PMC6085694

[B4] Cheng H. C. Varshney A. (2017). Volume segmentation using convolutional neural networks with limited training data, in IEEE International Conference on Image Processing (Beijing), 590–594. 10.1109/ICIP.2017.8296349

[B5] Çiçek Ö. Abdulkadir A. Lienkamp S. S. Brox T. Ronneberger O. (2016). 3d u-net: learning dense volumetric segmentation from sparse annotation, in International Conference on Medical Iimage Computing and Computer-Assisted Iintervention (Athens: Springer), 424–432. 10.1007/978-3-319-46723-8_49

[B6] Graham S. Vu Q. D. Raza S. E. A. Azam A. Tsang Y. W. Kwak J. T.. (2019). Hover-net: Simultaneous segmentation and classification of nuclei in multi-tissue histology images. Med. Image Anal. 58:101563. 10.1016/j.media.2019.101563 31561183

[B7] He K. Zhang X. Ren S. Sun J. (2016). Deep residual learning for image recognition, in IEEE Conference on Computer Vision and Pattern Recognition (Las Vegas, NV), 770–778. 10.1109/CVPR.2016.90

[B8] Kingma D. P. Ba J. (2014). Adam: A method for stochastic optimization. arXiv preprint arXiv:1412.6980.

[B9] Kirillov A. He K. Girshick R. Rother C. Dollár P. (2019). Panoptic segmentation, in Proceedings of the IEEE Conference on Computer Vision and Pattern Recognition (Long Beach, CA), 9404–9413. 10.1109/CVPR.2019.00963

[B10] Kumar N. Verma R. Sharma S. Bhargava S. Vahadane A. Sethi A. (2017). A dataset and a technique for generalized nuclear segmentation for computational pathology. IEEE Trans. Med. Imaging 36, 1550–1560. 10.1109/TMI.2017.2677499 28287963

[B11] Litjens G. Kooi T. Bejnordi B. E. Setio A. A. A. Ciompi F. Ghafoorian M.. (2017). A survey on deep learning in medical image analysis. Med. Image Anal. 42, 60–88. 10.1016/j.media.2017.07.005 28778026

[B12] Lucchi A. Li Y. Fua P. (2013). Learning for structured prediction using approximate subgradient descent with working sets, in IEEE Conference on Computer Vision and Pattern Recognition (Portland, OR), 1987–1994. 10.1109/CVPR.2013.259

[B13] Lucchi A. Smith K. Achanta R. Knott G. Fua P. (2011). Supervoxel-based segmentation of mitochondria in EM image stacks with learned shape features. IEEE Trans. Med. Iimaging 31, 474–486. 10.1109/TMI.2011.2171705 21997252

[B14] Paszke A. Gross S. Massa F. Lerer A. Bradbury J. Chanan G.. (2019). Pytorch: An imperative style, high-performance deep learning library, in Advances in Neural Information Processing Systems (Vancouver, BC), 8024–8035.

[B15] Peng J. Wang Y. (2021). Medical image segmentation with limited supervision: a review of deep network models. IEEE Access 9, 36827–36851. 10.1109/ACCESS.2021.3062380

[B16] Peng J. Yuan Z. (2020). Mitochondria segmentation from EM images via hierarchical structured contextual forest. IEEE J. Biomed. Health Inform. 24, 2251–2259. 10.1109/JBHI.2019.2961792 31871001

[B17] Qin X. Zhang Z. Huang C. Dehghan M. Zaiane O. R. Jagersand M. (2020). U2-net: going deeper with nested u-structure for salient object detection. Pattern Recogn. 106:107404. 10.1016/j.patcog.2020.107404

[B18] Ronneberger O. Fischer P. Brox T. (2015). U-net: convolutional networks for biomedical image segmentation, in International Conference on Medical Image Computing and Computer-Assisted Intervention (Munich: Springer), 234–241. 10.1007/978-3-319-24574-4_28

[B19] Seo J. H. Agarwal E. Chae Y. C. Lee Y. G. Garlick D. S. Storaci A. M.. (2019). Mitochondrial fission factor is a novel myc-dependent regulator of mitochondrial permeability in cancer. EBioMedicine 48, 353–363. 10.1016/j.ebiom.2019.09.017 31542392 PMC6838406

[B20] Shelhamer E. Long J. Darrell T. (2017). Fully convolutional networks for semantic segmentation. IEEE Trans. Pattern Anal. Mach. Intell. 39, 640–651. 10.1109/TPAMI.2016.2572683 27244717

[B21] Wei D. Lin Z. Franco-Barranco D. Wendt N. Liu X. Yin W.. (2020). Mitoem dataset: large-scale 3d mitochondria instance segmentation from EM images, in International Conference on Medical Image Computing and Computer-Assisted Intervention (Lima: Springer), 66–76. 10.1007/978-3-030-59722-1_7 PMC7713709 33283212

[B22] Xiao C. Chen X. Li W. Li L. Wang L. Xie Q.. (2018). Automatic mitochondria segmentation for em data using a 3d supervised convolutional network. Front. Neuroanat. 12:92. 10.3389/fnana.2018.00092 30450040 PMC6224513

[B23] Yuan Z. Ma X. Yi J. Luo Z. Peng J. (2021). HIVE-Net: Centerline-aware hierarchical view-ensemble convolutional network for mitochondria segmentation in EM images. Comput. Methods Prog. Biomed. 2020:105925. 10.1016/j.cmpb.2020.105925 33508773

[B24] Yuan Z. Yi J. Luo Z. Jia Z. Peng J. (2020). EM-Net: Centerline-aware mitochondria segmentation in em images via hierarchical view-ensemble convolutional network, in 2020 IEEE 17th International Symposium on Biomedical Imaging (ISBI) (Iowa, IA), 1219–1222. 10.1109/ISBI45749.2020.9098328

[B25] Zhang Y. Ying M. T. Chen D. Z. (2019). Decompose-and-integrate learning for multi-class segmentation in medical images, in International Conference on Medical Image Computing and Computer-Assisted Intervention (Shenzhen: Springer), 641–650. 10.1007/978-3-030-32245-8_71

